# Equity in the Early Pain Management of Long Bone Fractures in Black vs White Patients: We Have Closed the Gap

**DOI:** 10.5811/westjem.18531

**Published:** 2024-08-01

**Authors:** Dietrich Jehle, Krishna K. Paul, Stanley Troung, Jackson M. Rogers, Blake Mireles, John J. Straub, Georgiy Golovko, Matthew M. Talbott, Ronald W. Lindsey, Charles P. Mouton

**Affiliations:** * University of Texas Medical Branch, Department of Emergency Medicine, Galveston, Texas; † University of Texas Medical Branch, Department of Pharmacology and Toxicology, Galveston, Texas; ‡ University of Texas Medical Branch, Department of Orthopaedic Surgery and Rehabilitation, Galveston, Texas; § University of Texas Medical Branch, Department of Family Medicine, Galveston, Texas

## Abstract

**Introduction:**

Patients with long bone fractures often present to the emergency department (ED) with severe pain and are typically treated with opioid and non-opioid analgesics. Historical data reveals racial disparities in analgesic administration, with White patients more likely to receive analgesics. With the diversifying US population, health equity is increasingly crucial. In this study we aimed to evaluate the early administration of opioid and non-opioid analgesia among Black and White patients with long bone and femur fractures in EDs over different time frames using a substantial database.

**Methods:**

We retrospectively extracted Information from 57 US healthcare organizations within the TriNetX database, encompassing 95 million patients. The ED records from 2003–2023 were subjected to propensity score matching for age and gender. We focused on four cohorts: two comprising Black and White patients diagnosed with long bone fractures, and another two with Black and White patients diagnosed solely with femur fractures. We examined analgesic administration rates over 20 years (2003–2023) at five-year intervals (2003–2008; 2008–2013; 2013–2018; 2018–2023), and further analyzed the rates for the most recent two-year period (2021–2023).

**Results:**

Disparities in analgesic administration significantly diminished over the study period. For patients with long bone fractures (1,095,052), the opioid administration gap narrowed from 6.3% to 1.1%, while non-opioid administration disparities reduced from 4.4% to 0.3%. Similar trends were noted for femur fractures (265,181). By 2021–2023, no significant differences in analgesic administration were observed between racial groups.

**Conclusion:**

Over the past 20 years, the gap in early administration of opioid and non-opioid analgesics for Black and White patients presenting with long bone fractures or femur fractures has been disappearing.

Population Health Research Capsule What do we already know about this issue?

*Effective pain management for long bone fractures is crucial. Disparities in analgesic prescribing based on race highlight the need for health equity.*

What was the research question?

*Are there inequities in administration of analgesia for long bone fractures of Black vs White patients?*

What was the major finding of the study?

*From 2003 to 2023, the opioid administration gap narrowed from 6.3% (P < 0.001, CI 0.65–0.75) to 0.2% (P = 0.78, CI 0.98–1.03).*

How does this improve population health?

*Racial disparities are a significant barrier to equitable treatment, and this study helps shed light on the current state of healthcare.*

## INTRODUCTION

### Background

Patients with long bone fractures routinely present to the emergency department (ED) with severe pain, requiring effective pain management strategies. Over 178 million new fractures were recorded worldwide in 2019, and approximately 60% of those were long bone fractures.^1^ ^,^ ^2^ Standard management principles use non-opioid and opioid analgesics to treat the severe pain often associated with long bone fractures.^3^ There have been findings suggesting that not all patients’ pain was being treated equitably. A national study published in 2008 that covered a 13-year period showed that non-Hispanic White patients were 8% (31% vs 23%) more likely than Black patients to receive opioids for pain-related conditions including nephrolithiasis and long bone fractures.^4^ Several subsequent studies have shown the same trend, showing that non-Hispanic White patients were more likely to be treated with pain medications than other races for long bone fractures.^4^ ^–^ ^10^

In contrast, some recent smaller studies have reported no statistically significant racial differences in analgesic prescribing for patients with long bone fractures.^11^ ^–^ ^14^ For example, a study published in 2021 with 6,441 pediatric visits showed there was no significant difference in the rate of opioids prescribed to children who were Black, Hispanic, or other race vs non-Hispanic White children who were treated for long bone fractures at multiple hospitals from 2012–2019.^15^

As the population of the United States grows, its diversity is expected to increase as well. Minority groups are projected to exceed over 50% of the US population by 2044.^16^ Thus, health equity and awareness of differential treatment based on race or ethnicity becomes more significant. The fundamental ideal in medicine is to ensure a healthcare system that does not produce inequitable health treatment and outcomes based on an individual’s demographic characteristics.

### Goals of Investigation

Our primary objective was to investigate potential racial inequalities in the administration of opioid and non-opioid analgesics for patients with long bone and femur fractures in the ED.

## METHODS

### Study Design and Setting

This study employed a propensity-matched, retrospective design to evaluate a large national database, TriNetX, over various time periods. Using the “United States Collaborative Network” within the platform, containing de-identified electronic health records of approximately 95 million patients from 57 healthcare organizations, we created two sets of cohorts on December 27, 2022. These organizations are largely tertiary academic centers and their satellite facilities. There is representation from all geographic regions of the United States.

### Cohort Selection

We selected patients from all age groups. To protect patient privacy, those who were ≥90 years of age were grouped as 90 within the TrinetX database. In the first set of cohorts studied, Cohort 1.1 contained Black patients with long bone fractures, while Cohort 1.2 contained White patients with long bone fractures. Long bone fractures are defined using the International Classification of Diseases, 10^th^ Rev, procedure coding system (ICD-10) for diagnosis of fractures of the shaft of the tibia and femur, lower end of ulna, upper end of radius, shaft of ulna and fibula, lower end of radius, forearm, and shoulder and upper arm. The ICD-10 codes are listed in Table 1. Of note, multiple fracture diagnoses may be present in one visit. In the second set of cohorts, Cohort 2.1 had Black patients with only femur fractures and Cohort 2.2 had White patients with only femur fractures. In these cohorts, patients had come through emergency department services (CPT:1013711) within the prior 20 years.

**Table 1.** International Classification of Diseases, 10^th^ Rev, for fracture diagnosis in database.

Fracture diagnosis	ICD-10-CM	No. of fractures in database
Shoulder and upper arm	S42	1,073,481
Forearm	S52	1,072,733
Lower radius	S52.5	679,479
Femur	S72	597,633
Lower ulna	S52.6	346,093
Shaft of tibia	S82.2	290,731
Upper radius	S52.1	259,067
Shaft of ulna	S52.2	228,245
Unspecified shaft of fibula, initial encounter, closed	S82.409A	57,426

*ICD-10-CM*, International Classification of Diseases, 10^th^ Rev, Clinical Modification.

We further explored the cohorts by looking at a subgroup analysis over five-year intervals ranging from 2003–2008, 2008–2013, 2013–2018, and 2018–2023. These rounded cutoffs were chosen for ease of interpretation. We analyzed each interval from January of the starting year to January 1 of the final year. Both long bone and femur fracture cohorts were chosen in part to control for confounders in cases with multiple fractures, but also because femur fractures comprise a more homogeneous group that more consistently needs analgesia. Institutional review board approval was not required for this study, as TriNetX provides data that has been de-identified, which restricts access to protected health information (for users of the database).^17^

### Measures

Demographics included self-reported gender race, ethnicity, and marital status, which map to Health Level 7 administrative standards. Gender was coded as male or female. Race and ethnicity were recoded into Hispanic, non-Hispanic White, and non-Hispanic Black.

### Outcomes

From each cohort, two different outcomes were evaluated: treatment with opioid analgesics (VA:CN101) and treatment with non-opioid analgesics (VA:CN103). The administration data presented was binary: whether the patient had received any amount of analgesia or not. The time window was adjusted for the outcome to occur on the same day or up to one day from the index event for each cohort. Patients were excluded from the cohort if they received the outcome prior to the visit, such as those who may have had a documented acute or chronic opioid/non-opioid prescription prior to arrival.

### Statistical Analysis

Using the TriNetX database, a 1:1 propensity score match was produced with linear and logistic regression. We used greedy nearest neighbor matching with tolerance of 0.1 and difference between propensity ≤0.1.^18^ Balance on covariates was assessed using standardized mean difference, and absolute values of >0.1 were considered positive for residual imbalance. The TriNetX platform uses input matrices of user-identified covariates and conducts linear and logistic regression analysis to obtain propensity scores for individual subjects. TriNetX randomizes the order of rows to eliminate bias resulting from the nearest neighbor algorithms. This study methodology has been previously validated.^17^

We compared cohorts before and after propensity matching. Propensity matching was done through the “Balance Cohorts” tool in TriNetX to control for age at the diagnosis of the fracture and gender. There were statistically significant differences in the demographics for all compared cohorts prior to propensity matching. Demographics before and after propensity matching for the cohorts with long bone fractures or femur fractures in the last 20 years are shown in Table 2. Due to recent increases in societal awareness for differential treatment based on racial disparities, a second subgroup analysis was also completed on January 1, 2023, to analyze the same outcomes (opioid analgesics and non-opioid analgesics on the same day to one day after emergency care) for Black compared to White patients over the most recent two years (2021–2023).

**Table 2.** Demographics for Black patients vs White patients from 2003–2023 before and after propensity score matching.

Long bone fractures	Black patients before	White patients before	Black patients after	White patients after
Total patients	172,411	901,998	172,411	172,411
Age at index	34.4 +/− 24.2	43.8 +/− 28.5	34.4 +/− 24.2	34.4 +/− 24.2
Female	72,411 (42.0%)	462,246 (51.2%)	72,411 (42.0%)	72,411 (42.0%)
Male	99,904 (57.9%)	430,113 (47.7%)	99,904 (57.9%)	99,904 (57.9%)
**Femur fractures**	**Black patients before**	**White patients before**	**Black patients after**	**White patients after**
Total patients	39,360	220,840	39,360	39,360
Age at index	45.7 +/− 25.5	62.5 +/− 24.9	45.7 +/− 25.5	45.7 +/− 25.5
Female	16,899 (42.9%)	126,750 (57.4%)	16,899 (42.9%)	16,899 (42.9%)
Male	22,441 (57%)	91,567 (41.5%)	22,441 (57%)	22,441 (57%)

We used the measure-of-association tool in TriNetX to perform univariate analysis where risk ratio (RR), 95% confidence interval (CI), and probability values (*P*) were calculated to show comparisons of outcomes for the time intervals corresponding to each cohort studied. The data was reported as RRs with 95% CI from the final analysis, which was completed on December 29, 2022. Statistical significance was set at a two-sided alpha <0.05.

## RESULTS

### Characteristics of Study Subjects

In this study we analyzed 94,990,854 patients from 57 healthcare organizations in the United States Collaborative Network database in TriNetX. There were 2,477,404 patients identified with long bone fractures (272,690 Black and 1,716,194 White) and 597,833 patients with only femur fractures (67,905 Black and 431,613 White) before restricting to ED visits. After restricting the study to the ED, 175,354 Black and 919,698 White patients were found with long bone fractures. Using the five-year intervals from the subgroup analysis of long bone fractures, the number of Black patients found were 9,917 (2003–2008); 27,294 (2008–13); 67,767 (2013–18); and 82,008 (2018–23) while the number of White patients found were 44,864 (2003–2008); 147,130 (2008–13); 340,320 (2013–18); and 459,479 (2018–23), respectively.

For patients who came through the ED in the prior 20 years with femur fractures, there were 40,084 Black and 225,097 White patients. Looking at the same five-year intervals for the subgroup analysis of femur fractures, there were 2,092 (2003–2008) 5,827 (2008–13); 15,521 (2013–18); and 19,677 (2018–23) Black patients, while there were 9,301 (2003–2008); 33,098 (2008–13); 82,390 (2013–18); and 117,103 (2018–23) White patients, respectively. We defined race as “unknown” in 19% of the patient population. The geographic distribution of patients in the cohorts of ED patients with long bone fractures was 25% from the Northeast, 20% from the Midwest, 39% from the South, and 13% from the Western US.

### Main Results

**Figure 1.**
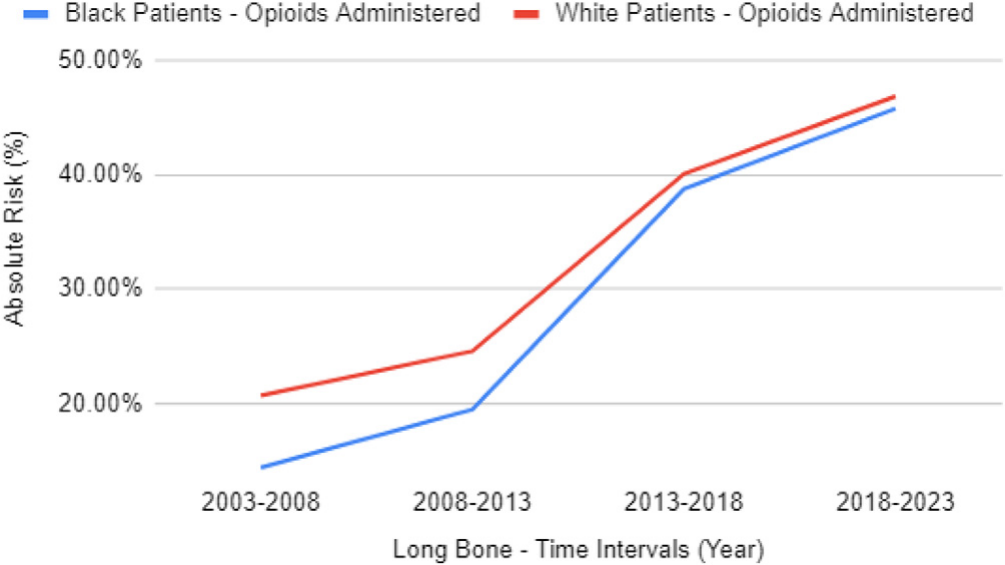
Rate of opioid administration for Black patients vs White patients with long bone fractures after propensity matching.

Opioid analgesia for patients with long bone fractures increased for both cohorts, shifting from 14.4% of Black and 20.7% (RR 0.697, 95% CI 0.647–0.751) of White patients between 2003–2008 to 45.8% of Black and 46.9% (RR 0.978, 95% CI 0.964–0.992) of White patients between 2018–2023. Similar increases are seen with non-opioid analgesia, going from 13.1% of Black and 17.5% of White patients (RR 0.751, 95% CI 0.692–0.815) between 2003–2008 to 42.0% for Black and 41.7% of White patients (RR 1.007, 95% CI 0.991–1.024) between 2018-2023. Additional data can be found in Table 3 and Table 4. The difference between opioid analgesics prescribed for Black vs White patients with long bone fractures has overall decreased from a significant 6.3% gap to 1.1% gap from the time intervals of 2003–2008 to 2018–2023, as seen in Figure 1. For patients with long bone fractures in the ED in the prior 20 years (80% are from the most recent 10 years), 37.7% of Black patients received opioid analgesia compared to 39.8% of White patients (RR 0.947, 95% CI 0.937–0.957), while 34% of Black patients received non-opioid analgesia compared to 35.6% of White patients (RR 0.955, 95% CI 0.944–0.967).

**Table 3.** Opioid analgesic administration for Black patients vs White patients from 2003–2023 after propensity score matching.

Long bone fractures	Black patients % (n)	White patients % (n)	RR (95% CI)
2003–2008	14.4% (997)	20.7% (1,346)	0.697 (0.647, 0.751)
2008–2013	19.5% (3,318)	24.6% (4,210)	0.792 (0.761, 0.824)
2013–2018	38.8% (14,250)	40.1% (15,392)	0.969 (0.952, 0.986)
2018–2023	45.8% (19,178)	46.9% (21,168)	0.978 (0.964, 0.992)
2021–2023	45.0% (7,223)	44.8% (7,643)	1.003 (0.981, 1.028)
2003–2023	37.7% (37,412)	39.8% (41,408)	0.947 (0.937, 0.957)
**Femur fractures**	**Black patients % (n)**	**White patients % (n)**	**RR (95% CI)**
2003–2008	17.8% (213)	22.6% (248)	0.786 (0.667, 0.926)
2008–2013	29.9% (839)	29.7% (806)	1.006 (0.928, 1.091)
2013–2018	54.1% (3,495)	53.0% (3,401)	1.021 (0.989, 1.055)
2018–2023	68.1% (5,196)	69.4% (5,511)	0.982 (0.961, 1.003)
2021–2023	69.6% (2,186)	70.1% (2,228)	0.993 (0.962, 1.026)
2003–2023	54.8% (9,726)	54.4% (9,777)	1.008 (0.989, 1.027)

*RR*, relative risk; *CI*, confidence interval; time intervals from initial Jan 1 to final Jan 1.

**Table 4.** Non-opioid analgesic administration for Black patients vs White patients from 2003–2023 after propensity score matching.

Long bone fractures	Black patients % (n)	White patients % (n)	RR (95% CI)
2003–2008	13.1% (886)	17.5% (1,072)	0.751 (0.692, 0.815)
2008–2013	19.0% (3,109)	23.6% (3,985)	0.807 (0.774, 0.841)
2013–2018	34.8% (12,004)	36.3% (13,774)	0.957 (0.939, 0.976)
2018–2023	42.0% (15,044)	41.7% (17,582)	1.007 (0.991, 1.024)
2021–2023	41.0% (5,473)	40.0% (6,270)	1.025 (0.996, 1.054)
2003–2023	34.0% (30,735)	35.6% (35,604)	0.955 (0.944, 0.967)
**Femur fractures**	**Black patients % (n)**	**White patients % (n)**	**RR (95% CI)**
2003–2008	16.3% (196)	18.7% (199)	0.876 (0.732, 1.047)
2008–2013	25.4% (695)	25.5% (722)	0.995 (0.910, 1.089)
2013–2018	45.1% (2,817)	45.0% (2,966)	1.004 (0.966,1.043)
2018–2023	58.6% (4,034)	59.0% (4,464)	0.994 (0.967, 1.021)
2021–2023	58.5% (1,668)	59.1% (1,798)	0.990 (0.948, 1.033)
2003–2023	46.3% (7,712)	45.3% (8,120)	1.023 (0.999,1.046)

*RR*, relative risk; *CI*, confidence interval; time intervals from initial Jan 1 to final Jan 1.

**Figure 2.**
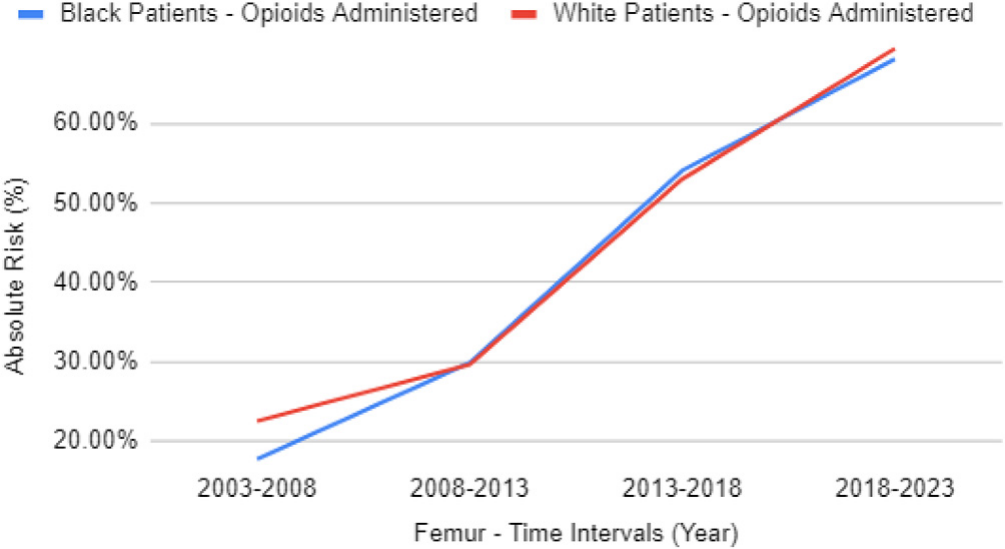
Rate of opioid administration for Black patients vs White patients with femur fractures after propensity matching.

Opioid analgesia for patients with femur fractures has been increasing for both cohorts, going from 17.8% of Black and 22.6% of White patients (RR 0.786, 95% CI 0.667–0.926) between 2003–2008 to 68.1% of Black and 69.4% of White patients (RR 0.982, 95% CI 0.961–1.003) between 2018–2023. This increase is also seen in non-opioid analgesia, increasing from 16.3% of Black and 18.7% of White patients (RR 0.876, 95% CI 0.732–1.047) between 2003–2008 to 58.6% of Black and 59% of White patients (RR 0.994, 95% CI 0.967–1.021) between 2018–2023. Additional data can be found in Table 3 and Table 4. The difference between opioid analgesics prescribed for Black vs White patients with femur fractures has overall decreased from a 4.8% gap to 1.3% gap from the time intervals of 2003–2008 to 2018–2023, as seen in Figure 2. For patients with femur fractures in the ED in the prior 20 years (80% are from the most recent 10 years), opioid analgesia was given to 54.8% of Black vs 54.4% of White patients (RR 1.008, 95% CI 0.989–1.027) while non-opioid analgesia was given to 46.3% of Black vs 45.3% of White patients (RR 1.023, 95% CI 0.999–1.046).

### Subgroup Analysis

In the subgroup analysis performed January 5, 2023, administration of opioid analgesics and non-opioid analgesics for Black patients was compared to White patients for long bone and only femur fractures during ED visits from 2021–2023. Before propensity matching was performed, a total of 229,395 patients were identified with long bone fractures and 62,761 patients with only femur fractures during this period. After propensity matching, there were a total of 69,668 patients with long bone fractures and 18,992 patients with only femur fractures during this period. The analysis after propensity matching showed that Black and White patients were both given comparable opioid analgesics (45.0% vs 44.8%, RR 1.00, 95% CI 0.980–1.028) and non-opioid analgesics (41.0% vs. 40.0%, RR 1.025, 95% CI 0.996–1.054) for long bone fractures during this time interval. When evaluating for only femur fractures, the findings were also comparable. Black and White patients were given equivalent opioid analgesics (69.6% vs 70.1%, RR 0.993, 95% CI 0.962–1.026) and non-opioid analgesics (58.5% vs 59.1%, RR 0.990, 95% CI 0.948–1.033).

### Comparison of Groups

The outcomes for prescribing of opioid analgesics and non-opioid analgesics for Black vs White patients presenting to the ED in all time intervals and subgroups were all collected on the same day or within 24 hours after the index event. During the period 2003–2008, White patients were prescribed significantly more opioid analgesics than Black patients; however, the gap has been decreasing, and between the years 2021–2023, we found no significant difference in the number of opioid analgesics prescribed for either the long bone fracture or femur fracture cohorts.

## DISCUSSION

The objective of this study was to investigate the relationship between racial disparities and the administration of both opioid and non-opioid analgesics following various long bone and femur fractures, and the trend over a 20-year time span to determine whether disparities have diminished. The sample included over 2.4 million patients across the US who were evaluated for pain associated with long bone fractures. Previous studies have concluded that the pain for these fractures is undermanaged for non-White patients.^6^ In the 2003–2008 cohort, the findings of previous studies were confirmed, that White non-Hispanic patients were previously administered analgesia significantly more often than other demographics.^4^ ^–^ ^8^ ^,^ ^10^ The results of this study suggest that the gap between analgesia administration rates has diminished. In fact, there was no statistical significance in opioids given to Black patients with long bone fractures over White patients over the most recent years (2021–2023). Results were similar with and without propensity matching of the given populations. The cohorts of femur fractures showed similar findings; however, the gap in administration of opioids appears to have narrowed in 2008 and has remained insignificant up to 2023.

Employing propensity matching by age and gender is important in this database, given the marked differences in the epidemiology of long bone and femur fractures across races. Notably, racial disparities in fracture incidence are evident across various age groups, from preschool to 60 years of age. Black males show a significantly higher incidence of fractures up to the age of 62, while Black females experience a modestly elevated rate of fractures until the age of 40. Furthermore, fractures attributed to violence are tenfold higher among Black individuals compared to other racial groups. Interestingly, despite possessing greater bone density, Black individuals, including both children and adults, exhibit an increased susceptibility to fractures across most non-fall-related injury mechanisms. This highlights the complexity of the factors influencing fracture risks and the significance of considering age, gender, and race in the analysis.^19^

This study demonstrates that, overall, opioid administration has increased over the past 20 years. Several factors contributed to this trend, including recent misrepresentations by drug companies about the risks associated with opioids,^20^ as well as policy initiatives from the Joint Commission on Accreditation of Healthcare Organizations which incentivized hospitals and physicians for more aggressive pain management. The results are consistent with a 2008 study that showed an increase in opioid use during times shortly after the launch of Joint Commission initiatives.^4^ In 1997, pain standards were developed through policies developed by the Joint Commission due to a need for organized pain assessment. The policies emphasized pain as the “5th vital sign,” which resulted in an upward trend for administration and prescription of opioid medications as treatment.^21^

Time to pain management became a core quality metric by which EDs were measured for multiple years and was linked to Medicare CMS reimbursement. Physicians and hospitals were evaluated based on their pain treatment practices, and financial incentives were provided for meeting certain criteria. This, in conjunction with increased societal awareness regarding disparities in healthcare, may have played a role in narrowing the gap in analgesia administration between Black and White patients. The combination of performance-based assessments and incentives, along with heightened public consciousness, likely contributed to changes in pain management practices that addressed previously existing disparities. There has been a more recent trend as a result of the opiate pandemic that shows overall opioid prescriptions are now decreasing to all patients.^14^

The results of this study have important implications for the acute management of patients with long bone fractures in the US. Inadequately treated pain has been found to be a major public health challenge in the US, and racial and ethnic minority groups have historically appeared to be at a high risk of receiving inadequate pain treatment in the ED.^4^ While studies as recent as 2020 have concluded that racial and ethnic minorities are less likely to receive analgesia for acute trauma,^10^ the results of this study show that the differential in treatment has disappeared. We incorporated a larger sample size that is approximately 10 times larger than any previous study on this topic, and through propensity matching some of the confounding variables were eliminated that may have skewed previous data. Future studies should aim to address other variables contributing to this decrease in disparity and incorporate more stratification of race and ethnicity to determine whether other disparities are present. Other possible relationships, such as rates of analgesic refusal by race, prehospital administration of analgesia, disparities in assessment of pain score by emergency clinicians, and language barriers should also be analyzed.

## LIMITATIONS

Because this was a retrospective study, causation between racial disparities and opioid administration could not be established. However, the size of this study – 2.4 million vs 157,000 - in conjunction with propensity matching, gave us greater power to identify differences in outcomes between groups compared to previous studies.^3^ ^,^ ^11^ ^–^ ^15^

In this propensity-matched retrospective study out of this national database, it becomes difficult to evaluate clinical details about each patient encounter such as pain scores, amount of analgesia, compliance to medications given, Emergency Severity Index acuity level, multitrauma, and information about the prescriber. This leads to lack of objectively measuring the effectiveness of the analgesia, whether additional treatment was needed after the initial analgesia, and, overall, a limitation in judging the effectiveness of the treatment itself. This study, however, was dedicated to a more short-term approach regarding the use of medications in the ED, not necessarily the effectiveness of post-encounter or medications prescribed upon discharge.

There may be an issue with granularity of the visit type and date in data collected from ICD 9/10-based systems. This difference should not be significant between the groups or lead to confounding. Approximately 80% of the patient population is from the past 10 years as many additional healthcare organizations have recently joined the TriNetX database. As a result, the dataset that includes all 20 years is skewed toward more recent patients. This effect is minimized by looking at two- and five-year periods.

Additionally, the inability to obtain insurance information for each patient may have posed a particular challenge as this can account for certain biases that affect financial access to treatment and may not be measurable on this scale.^7^ Our aim was to eliminate confounding variables such as this, and further information on insurance per patient may provide data allowing us to better understand the differences being measured in these sets after propensity matching. Although propensity matching was performed for demographic information, there may be pre-existing medical conditions that impact pain severity and administration of analgesia for which the study did not control. In addition, race was not known for approximately one-fifth of the patient population.

In the TriNetX database, the identities or designations of the healthcare organizations and their respective sites are not disclosed since the data is de-identified. Consequently, we could not consider any clustering by hospital. This limitation is significant as there may be inherent differences in the population characteristics across various hospital systems or sites, which could influence the administration patterns of analgesia and potentially introduce bias into the results. Moreover, our database does not provide information that allowed us to discern whether analgesics were administered to individual patients in the prehospital setting, which may have served as an additional confounding factor. There is evidence indicating that prior to 2020, disparities existed in the administration of pain medication in the prehospital setting. This was observed in cases involving both non-traumatic and traumatic painful conditions, where the probability of Black patients receiving pain medication was lower when compared to White patients.^22^

## CONCLUSION

This retrospective analysis provides evidence from healthcare centers across the US that there is no longer a significant difference in the administration of opioid and non-opioid analgesics between Black and White patients diagnosed with long bone and femur fractures.
